# Recent Advance in the Relationship between Excitatory Amino Acid Transporters and Parkinson's Disease

**DOI:** 10.1155/2016/8941327

**Published:** 2016-02-14

**Authors:** Yunlong Zhang, Feng Tan, Pingyi Xu, Shaogang Qu

**Affiliations:** ^1^Department of Blood Transfusion, The Fifth Affiliated Hospital, Southern Medical University, Guangzhou, Guangdong 510900, China; ^2^Department of Traditional Chinese Medicine, College of Medicine, Xiamen University, Xiamen, Fujian 361102, China; ^3^Department of Neurology, Foshan Hospital of Traditional Chinese Medicine, Guangzhou University of Chinese Medicine, Foshan, Guangdong 528000, China; ^4^Department of Neurology, The First Affiliated Hospital of Guangzhou Medical University, Guangzhou, Guangdong 510080, China

## Abstract

Parkinson's disease (PD) is the most common movement disorder disease in the elderly and is characterized by degeneration of dopamine neurons and formation of Lewy bodies. Glutamate is the major excitatory neurotransmitter in the central nervous system (CNS). If glutamate is not removed promptly in the synaptic cleft, it will excessively stimulate the glutamate receptors and induce excitotoxic effects on the CNS. With lack of extracellular enzyme to decompose glutamate, glutamate uptake in the synaptic cleft is mainly achieved by the excitatory amino acid transporters (EAATs, also known as high-affinity glutamate transporters). Current studies have confirmed that decreased expression and function of EAATs appear in PD animal models. Moreover, single unilateral administration of EAATs inhibitor in the substantia nigra mimics several PD features and this is a solid evidence supporting that decreased EAATs contribute to the process of PD. Drugs or treatments promoting the expression and function of EAATs are shown to attenuate dopamine neurons death in the substantia nigra and striatum, ameliorate the behavior disorder, and improve cognitive abilities in PD animal models. EAATs are potential effective drug targets in treatment of PD and thus study of relationship between EAATs and PD has predominant medical significance currently.

## 1. Introduction

Glutamate is the predominant excitatory neurotransmitter in the central nervous system (CNS). It is released from presynaptic glutamatergic neurons and activates the ionotropic and metabotropic glutamate receptors located on the postsynaptic neurons. Glutamate in the synaptic cleft is kept in a low concentration, while excessive glutamate will overstimulate the glutamate receptors and mediate the excitotoxic effects on the CNS [1, 2]. Previous studies have revealed that glutamate excitotoxicity induces the dopamine (DA) neurons death, movement disorder, and cognitive impairment, and thus glutamate excitotoxicity plays an important role in the pathogenesis of Parkinson's disease (PD) [3, 4]. Glutamate uptake in the synaptic cleft is performed by the excitatory amino acid transporters (EAATs, also known as high-affinity glutamate transporters). Five mammalian EAATs have been characterized: GLAST (glutamate/aspartate transporter, also called EAAT1), GLT-1 (glutamate transporter-1, also called EAAT2), EAAC1 (excitatory amino acid carrier-1, also called EAAT3), EAAT4, and EAAT5 [5–10]. Among these, astrocytic GLT-1 and, to a lesser extent, GLAST are mainly responsible for the glutamate uptake and mitigate excitotoxicity. Increasing evidences suggest that dysfunctional EAATs expressions are found in PD patients and models [11–14], and, moreover, DA neurons expressing EAAC1 are preferentially affected by EAATs dysfunction and* in vitro* studies show that application of EAATs substrate inhibitor is preferentially toxic for DA neurons by lowering their resistance threshold to glutamate excitotoxicity [15]. However, it is unclear whether decreased EAATs expression is the consequence or the cause of PD. Recently Assous et al. report that single unilateral administration of EAATs inhibitor in the substantia nigra mimics several PD features, as they find DA neurons death and axons dystrophy in the substantia nigra and striatum, and the motor disorder appears when DA neurons loss exceeds 50% [16]. Thus far this is a solid evidence which supports that dysfunctional EAATs are linked to the pathogenesis of PD.

In this review, we outline the recent advance of structure, function, and distribution of EAATs in the CNS, we also highlight the glutamate excitotoxicity in the pathogenesis of PD and the role of dysfunctional EAATs in the excitotoxicity, and we show the related finding in treatment of PD by upregulating EAATs in recent years.

## 2. Structure and Function of EAATs

**Figure 1:**
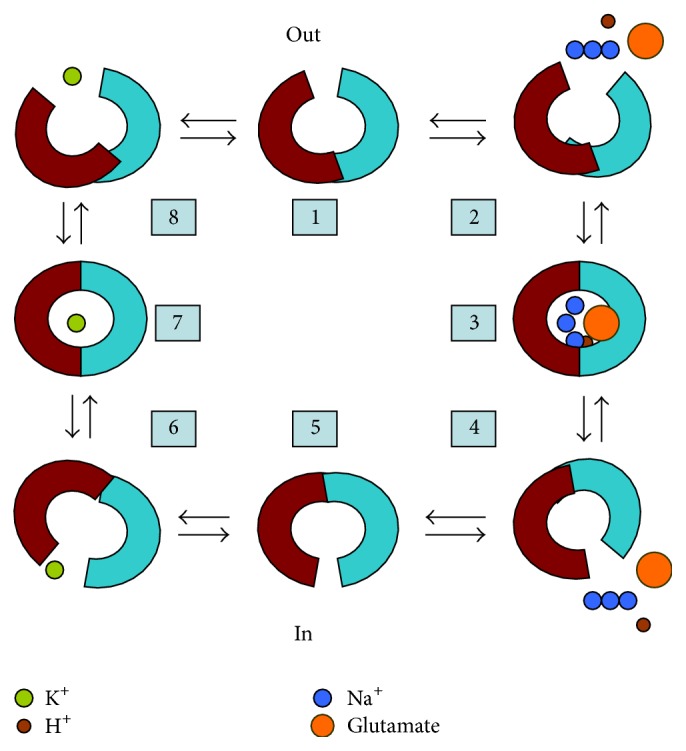
The mode of EAATs action. In the normal condition, the outward facing empty glutamate transporter (1) binds with glutamate, H^+^, and 3 Na^+^ (2) and turns into the fully loaded carrier state (3). Followed by releasing the substrates at the intracellular face (4), the transporter turns into the inward facing empty state (5). Thereafter, K^+^ binds to the inward facing transporter (6) and turns into the K^+^ loaded transporter state (7). Afterwards, the transporter releases K^+^ at the extracellular face (8) and translocates back into the outward facing empty glutamate transporter state (1).

As stated previously, these five mammalian EAATs have been cloned and characterized [5–10], and they share nearly 50–60% of homologous sequences [17]. In 2004, Yernool et al. revealed the crystal structure of prokaryotic aspartate transporter Glt_Ph_ from* Pyrococcus horikoshii*, which is the homologue of the glutamate transporters [18]. The transport domain for each Glt_Ph_ subunit is composed of eight transmembrane (TM) domains TM1–TM8 and two opposite-facing helical hairpins HP1 and HP2 [18]. Glutamate or aspartate transport process is operated via an “alternate access mechanism”; the mode of EAATs action is shown in Figure 1. Currently, the crystal structures of Glt_Ph_ at several different phases have been revealed. Briefly, these phases contain the substrate binding phase (PDB ID 1XFH), Glt_Ph_ binding with TBOA (PBD 2NWW), and Glt_Ph_ is trapped in the inward facing state (PDB ID 3KBC), Glt_Ph_ with two protomers in an inward facing state and the third in an intermediate conformation between the outward and inward facing states (PDB ID 3V8G), and also the newly reported Glt_Ph_ in the cation-only bound site (PDB ID 4P1A) [18–22]. These different phases are significant for further study of the structure and function of eukaryotic glutamate transporter.

EAATs terminate the glutamate excitatory synaptic transmission via uptake of glutamate in the synaptic cleft. In addition, through clearing the excessive glutamate in the synaptic cleft, EAATs could modulate the location of metabotropic glutamate receptor within the synapse, maintain the most effective distribution of glutamate, and keep the accurate neurotransmission in the synapse [23, 24]. Furthermore, EAATs can promote the astrocytes near the glutamatergic synapse releasing glutamine rapidly and provide the neurons with glutamine for synthesis of glutamate and *γ*-aminobutyric acid (*γ*-GABA) [23–25]. Glutamate transporters are also involved in regulating learning, memory, and the motor behavior [25–29]. Moreover, EAATs also provide cysteine or glutamate for the synthesis of glutathione and some other proteins [30].

Among these five subtypes, GLAST is abundantly expressed in the Bergmann glial cells in the molecular layer of the cerebellum, and it is also expressed in the spinal cord, forebrain, inner ear, and retina [31]. GLT-1 is widely distributed in the CNS and is mainly expressed in astrocytes in the forebrain, cerebral cortex, hippocampus, and other regions. Besides, GLT-1 is also expressed in the neurons in the development stage [7, 32, 33]. Recently, C-terminal splice variants of GLT-1 are found and among these splice variants GLT-1a, GLT-1b (also called GLT-1v), and GLT-1c are closely related to the neurodegenerative diseases [34]. They are mainly expressed in the neurons and also in astrocytes and microglia, and their distribution in different cell types is complementary [34]. GLT-1 is responsible for clearing nearly 90% of glutamate in the synaptic cleft. Using conditional knockout model, Petr et al. find that when astrocytic GLT-1 is eliminated, glutamate uptake in the forebrain synaptosomes shows no significant changes [35]. However, when GLT-1 is deleted in neurons, the glutamate uptake in the synaptosomes is decreased significantly [35]. This study reveals that lowly expressed GLT-1 in the neurons is responsible for the most of glutamate uptake in the forebrain synaptosomes; in addition, astrocytic and neuronal GLT-1 possibly take on different roles in the synaptic excitatory neurotransmission [35].

EAAC1 is mainly expressed in the postsynaptic membrane of neurons and is abundantly distributed in the hippocampal CA1–CA4 region and cortical pyramidal cell layers. Studies showed that EAAC1 is highly expressed in the hippocampus of young adult rat and per gram of tissue containing about 0.013 mg of EAAC1 [36]. EAAT4 is limitedly expressed in the Purkinje cells in the cerebellar molecular layer and EAAT5 is limitedly expressed in the neurons and astrocytes in the retina, and it is also expressed in the photoreceptors, bipolar cells, and amacrine cells [10, 37]. Recent studies also show that EAAT4 and EAAT5 are also expressed in type I and II vestibular hair cells [38].

## 3. Excitotoxicity in the Pathogenesis of Parkinson's Disease

### 3.1. Mechanism of Excitotoxicity in PD

PD is a common chronic neurodegenerative disease in elderly and is characterized by the progressive degeneration of DA neurons in the pars compacta of substantia nigra (SNpc) and the formation of Lewy bodies. Besides, glutamate excitotoxicity also participates in the pathogenesis of PD. Impaired motor coordination and dyskinesias in PD have been shown to be closely linked to the increase of glutamate levels within the basal ganglia [39–43]. Dopaminergic denervation also induces dysfunctional corticostriatal glutamate release, and this circuitry imbalance contributes to further DA neurons loss in substantia nigra [44]. In addition, depression, dementia, and the nonmotor symptoms in the pathogenesis of PD attract much more attention nowadays [45–49]. Dysfunction of glutamate metabolism is also involved in the cognitive impairment in PD [26, 27].

Excessive glutamate in the synaptic cleft overstimulates the ionotropic and metabotropic glutamate receptors in the postsynaptic membrane and mediates excitotoxicity. Through overactivating the N-methyl-D-aspartate (NMDA) receptor, glutamate can induce intracellular Ca^2+^ overload, production of reactive oxygen species, and reactive nitrogen radicals, result in mitochondrial dysfunction, and thus lead to the neuronal death; through overactivating *α*-amino-3-hydroxy-5-methyl-4-isoxazole-propionic acid (AMPA) receptor and kainic acid (KA) receptor, glutamate can induce Na^+^ influx and acute osmotic swelling of nerve cells and mediate neuronal death [50–55]. Current studies also show that, except glutamate, other factors also could induce excitotoxicity in PD. Alpha-synuclein could downregulate the expression and function of NR2B-containing NMDA receptors [56]. Knockout of *α*-synuclein can weaken the decreased expression of NR2B-containing NMDA receptors mediated by rotenone and reduce the cortical neurons death [56]. Moreover, glutamate also stimulates group I metabotropic glutamate receptors (mGluR), activates the intracellular phospholipase C (PLC), and induces the hydrolysis of PLC into inositol triphosphate (IP3) and diacylglycerol (DAG), wherein IP3 can induce intracellular Ca^2+^ release and DAG can activate protein kinase C (PKC) and strengthen the calcium influx mediated by NMDA receptor and thus these effects induce neuronal death [57–59].

**Figure 2:**
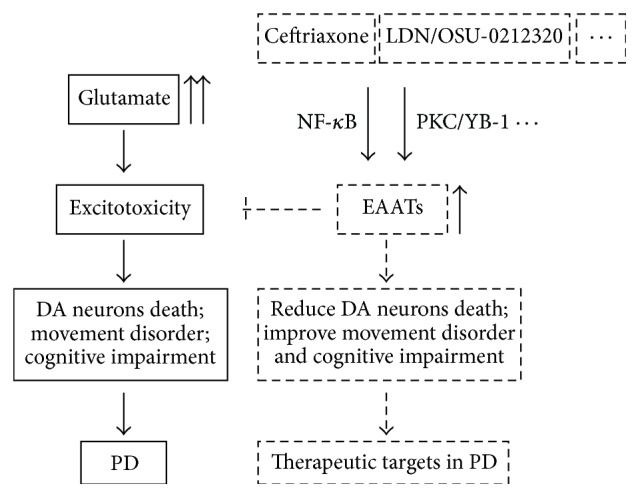
EAATs are therapeutic targets in PD. Excessive glutamate in the synaptic cleft overstimulates glutamate receptors in the postsynaptic membrane and mediates excitotoxicity. Glutamate excitotoxicity can induce the dopamine neurons death, movement disorder, and cognitive impairment, and thus it contributes to the pathogenesis of PD. Through upregulation of EAATs, ceftriaxone, LDN/OSU-0212320, and other drugs can reduce the DA neuron death in SNpc and striatum, improve the movement disorder and cognitive impairment in PD, and thus improve the PD progression. Thus, EAATs are therapeutic targets in PD.

Thus, accumulation of glutamate in the synaptic cleft and other pathological productions contribute to the pathogenesis of PD via different pathways and excitotoxicity is a key link in the process of PD (Figure 2).

### 3.2. Malfunction of EAATs Contributes to the Excitotoxicity in PD

Previously Ferrarese et al. study the glutamate uptake in platelet in PD patients and normal control, and they find that glutamate uptake in platelet in PD patients is reduced by 50% compared with control [60]. Moreover, the glutamate uptake reduction is related to the severity of PD [60]. Generally, dysfunction of glutamate transporters reduces the glutamate uptake, mediates the glutamate excitotoxicity and oxidative stress, damages the DA neurons in substantia nigra, and thus contributes to the pathogenesis of PD. We stated the relationship between EAATs and PD as follows.

#### 3.2.1. GLAST and PD

Some studies reported that GLAST protein expression is significantly decreased in the striatum at 1 week and 2 weeks after 6-hydroxydopamine (6-OHDA) lesion [11, 61]. Besides, GLAST mRNA expression is also decreased in the 6-OHDA lesion rats [41]. Considering that GLAST plays a lesser important role in the glutamate uptake, decreased GLAST expression possibly is a product in the pathogenesis of PD. Salvatore et al. use a novel GLAST inhibitor UCPH-101 ((2-amino-5,6,7,8-tetrahydro-4-(4-methoxyphenyl)-7-naphthalen-1-yl)-5-oxo-4H-chromene-3-carbonitrile) and GLT-1 inhibitor DHK to evaluate the role of GLAST and GLT-1 in PD. They find that GLAST-mediated reuptake component in striatum may be relatively minor, but it can be dramatically promoted when GLT-1 blockade increases extracellular glutamate levels [12]. Moreover, GLAST expression is also shown to be increased in striatum in 6-OHDA lesion rats [62]. As astrocytic GLT-1 is mainly responsible for glutamate reuptake, these studies reveal that GLAST may play a compensatory role when GLT-1 function is impaired in PD.

#### 3.2.2. GLT-1 and PD

As GLT-1 is mainly responsible for the glutamate uptake in the CNS, increasing evidences indicate the role of GLT-1 in PD. Impaired glutamate uptake and reduced GLT-1 expression are found in PD animal models constructed by 6-OHDA, 1-methyl-4-phenyl-1,2,3,6-tetrahydropyridine (MPTP), and 1-methyl-4-phenylpyridinium (MPP^+^) [11–14]. Using GLT-1 inhibitor blocks the glutamate uptake and reduces the expression of phosphorylated tyrosine hydroxylase and the synthesis of DA [12]. Also, DA denervation may trigger modulations in GLT-1 [63]. Thus, these results indicate that GLT-1 dysfunction plays a role in PD progression. To further explore the role of GLT-1 in PD, recently Assous et al. used the inhibitor of glutamate transporter PDC to inject in the unilateral SNpc of rats [16]. They find DA neurons death and axons dystrophy in the SNpc and striatum [16]. When the DA neurons loss exceeds 50%, the motor disorder appears in rats [16]. This study confirms that reduced glutamate uptake using glutamate transporter inhibitor leads to the DA neurons death and progressive parkinsonism symptoms, and this is a solid evidence which supports that reduced expression and function of glutamate transporter are involved in PD.

GLT-1 expression is also found to be increased in response to injury or excitotoxic insult [64, 65]. Besides, Massie et al. find that GLT-1 expression is increased in the striatum at 3 weeks and 12 weeks after 6-OHDA lesion [62]. Chronic inflammation is confirmed to contribute to the pathogenesis of PD [66]; Brothers et al. construct a PD model of chronic inflammation using lipopolysaccharide (LPS) and find reduced tyrosine hydroxylase expression in the substantia nigra and locus coeruleus and also the microglial activation [67]. Meanwhile, chronic inflammation increases extracellular glutamate concentration and GLT-1 expression in the hippocampal CA3 and the dentate gyrus regions [67]. GLT-1 expression and glutamate uptake are found obviously altered in PD models, and some studies report the related regulatory mechanism. Since DA neurons loss is the classical mechanism of PD, Vollbrecht et al. find that partial prefrontal cortex (PFC) dopamine depletion increases GLT-1 protein expression in the membrane and glutamate uptake, but dopamine depletion does not change the expressions of GLAST and EAAC1 [68]. These results suggest that dopamine depletion possibly promotes PFC astrocytic GLT-1 expression and activity via posttranslational modification [68]. It is clear that nuclear factor-*κ*B (NF-*κ*B) could bind with the GLT-1 promoter, and activation of NF-*κ*B signaling pathway is demonstrated to elevate GLT-1 transcription upon ceftriaxone treatment [69]. Our previous* in vitro* study indicates that neurotoxin MPP^+^ decreases GLT-1 expression in the membrane and induces astrocytic death via activation of NF-*κ*B/c-Jun N-terminal kinase (JNK)/c-Jun pathway [70].

#### 3.2.3. EAAC1 and PD

As mentioned previously, neuronal glutamate transporter EAAC1 is responsible for the glutamate uptake and cysteine uptake, and the latter is used to synthesize glutathione. As neuronal EAAC1 does not contribute to bulk glutamate uptake in the synaptic cleft, increasing studies focus on the antioxidative effects of EAAC1 in the pathogenesis of PD. EAAC1 deficiency mice show age-dependent loss of DA neurons in the SNpc, and nearly 40% of DA neurons are lost at 12 months' age [71]. Meanwhile, DA neurons loss is accompanied by increased nitrotyrosine formation, nitrosylated *α*-synuclein, and microglial activation [71]. Moreover, Kinoshita et al. find that cysteine in the mesencephalon is expressed in a diurnal variation manner and is accompanied by the diurnal fluctuations of EAAC1 protein expression [72]. MicroRNA-96-5p can downregulate EAAC1 expression, and intracerebroventricular administration of microRNA-96-5p inhibitor increases the levels of glutathione and EAAC1 expression in the substantia nigra, suggesting that suppressing microRNA-96-5p shows benefit in the progress of PD [72].

However, EAAC1 expression is also reported to be increased in PD patients and models. The* in situ* hybridization results performed by Plaitakis and Shashidharan in the brain in PD patients suggest that EAAC1 expression is increased in DA neurons and EAAC1 has a close correlation with PD [73]. Increased expression and function of EAAC1 are also found in PD animal models [11, 62]. GLT-1 blockade increased EAAC1 expression, suggesting the reciprocal regulation within different EAATs subtypes in PD progression [12]. Besides participating in glutathione homeostasis, the glutamate transported by EAAC1 also contributes to the neuronal metabolism and motor function. EAAC1 may participate in normal GABA neurosynthesis and plays a role in epilepsy [74].

As EAAT4 is limitedly expressed in the Purkinje cells in the cerebellum and EAAT5 is limitedly expressed in the retina, recently there is still lack of evidence on their contributions in PD.

These studies suggest that, in the process of PD, reduced EAATs expression induces the excessive extracellular glutamate and glutamate can damage the DA neurons via overstimulating the ionotropic and metabotropic glutamate receptors, while increased EAATs expression is involved in the compensation mechanism to reduce the glutamate excitotoxicity. The related regulatory mechanism of EAATs in PD still needs further study.

## 4. EAATs Are Potential Therapeutic Targets in PD

Upregulation of EAATs can weaken the excitotoxic damage to the CNS. Studies have shown that ceftriaxone can improve GLT-1 expression by activation of NF-*κ*B and reduce glutamate concentration in the synaptic cleft [69, 75, 76]. Through upregulation of GLT-1, ceftriaxone can reduce the DA neuron death in the SNpc and striatum and ceftriaxone can also improve the movement disorder and cognitive impairment in PD models (Figure 2) [77–80]. Besides, ceftriaxone can also improve the levodopa-induced dyskinesia via upregulation of GLT-1 [81]. In addition, ATP-sensitive potassium channel (KATP) opener iptakalim (IPT) can enhance the glutamate transporter function in astrocytes and reduce the extracellular glutamate, and thus IPT is also a prospective drug in treatment of PD [82]. The improvement of behavioral disorder and cognitive impairment by ceftriaxone and other drugs is closely linked to EAATs, because EAATs are also reported to participate in modulating learning, memory, and also motor function in human being and animal models [26–29]. MicroRNAs are a novel type of 19- to 22-nucleotide noncoding RNAs that modulate gene expression primarily at the posttranscriptional level. As stated previously, suppressing microRNA-96-5p can increase the levels of glutathione via promoting EAAC1 expression in PD [72]. Neuronal exosomes containing microRNA-124a and astrocyte-enriched microRNA-181 antagomir can increase GLT-1 expression and show benefit in forebrain ischemia and amyotrophic lateral sclerosis (ALS) animal models [83, 84]. These results suggest that microRNAs targeting EAATs are also possible therapeutic targets in PD. In addition to ceftriaxone, recently compound LDN/OSU-0212320 is also shown to promote GLT-1 expression and activity at the translational level via activation of PKC and subsequent Y-box-binding protein 1 (YB-1) specially [85] (Figure 2). N-acetylcysteine administration restores DA neurons loss, nitrosylated *α*-synuclein aggregation, and microglial activation in SNpc in EAAC1 deficiency mice [70]. Thus, N-acetylcysteine shows benefit via evaluation of glutathione in treatment of PD. The locomotor impairment is a common symptom in PD progression; Arnold and Salvatore indicate that short-term exercise can attenuate the locomotor impairment in aging rats via increasing nigral glial cell line-derived neurotrophic factor (GDNF) receptor, GFR-*α*1, and EAAC1 expression in conjunction with increased nigral tyrosine hydroxylase expression [86]. We previously found that ceftriaxone can improve the cell viability, glutamate uptake, and GLT-1 expression in the membrane in neurotoxin MPP^+^-treated astrocytes [70]. Furthermore, we explore the related mechanism and we find that, by inhibiting NF-*κ*B/JNK/c-Jun pathway, ceftriaxone enhances the astrocytes viability and attenuates the apoptosis mediated by neurotoxin MPP^+^, and ceftriaxone promotes the GLT-1 expression in the membrane and the glutamate uptake and thus reduces the glutamate excitotoxicity effects in MPP^+^-treated astrocytes [70].

In this review we indicate the structural and functional characters of EAATs and their distribution in the CNS, we also show that glutamate and other factors can mediate the excitotoxicity in PD, and we point out the related mechanism, respectively. Within these, decreased expression and function of EAATs play a predominant role in the excitotoxic damage in PD. According to previous studies and our work, we show that EAATs are novel therapeutic targets in PD and reveal that the structure, function, and regulation mechanism of EAATs will be a prospective research area in the clinical practice of PD.
